# Intrachromosomal colocalization strengthens co-expression, co-modification and evolutionary conservation of neighboring genes

**DOI:** 10.1186/s12864-018-4844-1

**Published:** 2018-06-13

**Authors:** Shuaibin Lian, Tianliang Liu, Shengli Jing, Hongyu Yuan, Zaibao Zhang, Lin Cheng

**Affiliations:** 1 College of Physics and Electronic Engineering, Xinyang Normal University, Xinyang, China; 2 College of Life Sciences, Xinyang Normal University, Xinyang, China; 3 Institute for Conservation and Utilization of Agro-bioresources in Dabie Mountains, Xinyang Normal University, Xinyang, China

**Keywords:** Neighboring gene pairs, Intrachromosomal colocalization, Evolutionary neighboring, Similarities

## Abstract

**Background:**

Gene order and location in chromosomes of species are non-random. Neighboring gene pairs tend to display some similarities, such as co-expression and co-modification. However, the contribution of linear proximity, spatial proximity, and evolutionary proximity to these similarities remain unclear, together with whether the presence of several types of proximity can strengthens the similarities.

**Results:**

In this study, we investigated the properties of three kinds of colocalized gene pairs: intrachromosomal colocalized gene pairs, always-neighboring gene pairs, and evolutionary neighboring gene pairs. Our analysis showed that (1) Different types of colocalized genes differentially contribute to co-expression, co-modifications and conservation across species; (2) Intrachromosomal colocalization can strengthen co-expression and co-modification of neighboring gene pairs and their conservation across species; (3) The combination of the three kinds of colocalization can lead to the strongest co-modification and is most strongly conserved across species. (4) Colocalized gene pairs are indicative of phylogenetic relationships and whole genome duplications (WGDs).

**Conclusions:**

These results provide valuable clues for future efforts to understand the characteristics of colocalized gene pairs and how the neighborhood affects their interactions.

**Electronic supplementary material:**

The online version of this article (10.1186/s12864-018-4844-1) contains supplementary material, which is available to authorized users.

## Background

Accumulating evidence indicates that gene order is not completely random in eukaryotic chromosomes. In humans, gene-rich chromosomes tend to occupy interior positions in the nucleus, whereas gene-poor chromosomes tend to be peripherally located [1]. Genes with similar expression levels tend to be clustered within the same genomic neighborhood [2–6], and these observations have been reported in many species of plants and animals [7–11]. In addition, genes expressed in specific tissues also tend to cluster on chromosomes [12]. For example, immune system genes and genes essential for viability are found in clusters in the mouse genome [13]. In the human genome, housekeeping genes also show strong clustering [14]. In the *Saccharomyces cerevisiae* genome, neighboring genes show similar patterns of histone modification [15]. Many factors are responsible for the co-expression of neighboring genes, such as sharing common promoter elements [16, 17], transcription factors [18, 19], and histones modifications [20, 21].

Moreover, co-expression of neighboring gene pairs may persist long after they separated during evolution [22], a phenomenon which may be ascribed to spatial colocalization despite the genes no longer being adjacent [23]. High-order folding of chromosomes increases the proximity of distant chromatin regions, creating the potential for genes in these regions to interact [24]. Recent evidence reveals that spatially colocalization of regions is linked with the regulation of gene expression [23, 25]. Functionally related genes tend to colocalize in the 3D space of the nucleus [25], and neighboring genes which have been separated still tend to show such spatial colocalization [23].

Notably, recent evidence indicates that the expression patterns of neighboring genes are correlated during evolution [26]. For example, gene expression in humans changes on a cluster-by-cluster basis, such that a change in the expression of any given focal gene can affect the expression of genes in its vicinity, though whether this implies natural selection for such clusters is unresolved. However, these clusters only include one type of neighboring gene pair, linear colocalization on the same chromosome. In fact, colocalization of gene pairs can be classified into three types: spatial colocalization (genes with interchromosomal or intrachromosomal colocalization), always-neighboring (genes which are neighbors now and have been in the evolutionary past), and evolutionary neighboring (genes which were separated in the evolutionary past but are now neighbors). It remains unclear whether evolutionary neighboring gene pairs caused by natural selection also tend to show similarities such as co-expression and co-modification.

**Fig. 1:**
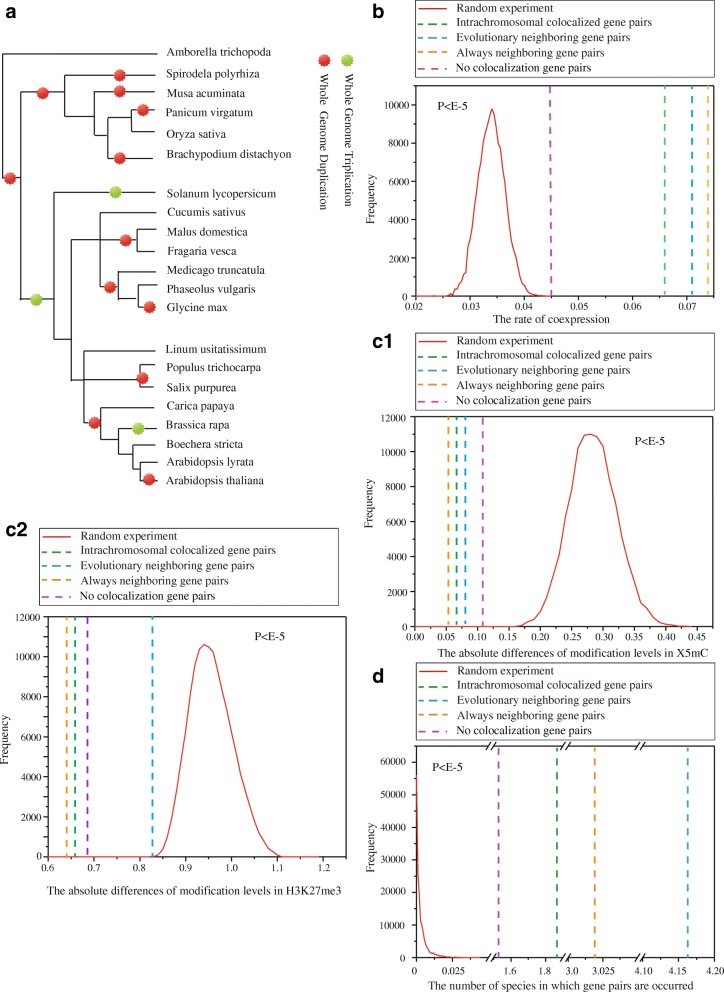
Different types of colocalized genes differentially contribute to co-expression, co-modification and conservation across species. **a** A phylogeny of species included in this study (partly adapted from [27]). Whole genome duplication and triplication events are marked according to the Plant Genome Duplication Database (PGDD) [28, 29]. **b** The frequency of co-expressed gene pairs of three colocalized gene pairs and no-colocalized genes in threshold 0.5. **c1** and **c2** The absolute difference in the modification levels of histone X5mC and H3K27me3, respectively, in three colocalized gene pairs and no-colocalized genes. **d** The number of species in which gene pairs of the three colocalizations and no-colocalizations are found. The red curves show the frequency distributions for 10,000 permuted randomizations of the same number of pairs as in the real data. Error bars were calculated by bootstrapping. Significance values calculated from the Mann–Whitney U test are shown

Consequently, it is interesting to investigate: 1) Whether these three kinds of colocalization can all contribute to similar co-expression and co-modification of gene pairs; 2) If so, how strong the contribution of each kind of colocalization is to the co-expression and co-modification; 3) Whether the combination of these colocalizations can strengthen similarities such as co-expression and co-modifications, as well as the strength of these effects in combination. To address these questions, we investigated the properties of intrachromosomal colocalized gene pairs, evolutionary neighboring gene pairs and always-neighboring gene pairs in the model species *Arabidopsis thaliana* in comparison with 20 other species, taking into account their phylogenetic relationships (Fig. 1a).

## Results

### Different types of colocalized genes differentially contribute to co-expression, co-modificcation and conservation across species

In order to investigate the special characteristics of the three types of gene pairs, we identified intrachromosomal colocalized gene pairs which were not linear neighbors (490 pairs, Additional file 1: Table S1), always-neighboring gene pairs without intrachromosomal colocalization (16,456 pairs, Additional file 2: Table S2), and evolutionary neighboring gene pairs without intrachromosomal colocalization (6205 pairs, Additional file 3: Table S3) from gene order data generated for *Arabidopsis thaliana* and 20 other species (Fig. 1a) (see Methods for details). We analyzed the expression profiles, histone modification levels, and conservation of these genes pairs across species. To ensure that the phenomena we observed were not due to chance, we compared our results with an analysis of an equal number of randomized gene pairs as well as no colocalization gene pairs. The two analyses should be similar if the phenomena are due to chance rather than being intrinsic characteristics of colocalized gene pairs; we repeated the random experiments 10,000 times to confirm the statistical significances of our results. Similarly, the similarities of colocalized genes should be significantly higher than no colocalization genes.

We first investigated the strength relationship of co-expression of gene pairs by computing the Pearson correlation coefficient using gene expression data from the TAIR database. We used a correlation threshold of 0.5 and 0.1 to judge high correlation or low correlation of gene pairs respectively. In terms of threshold of 0.5, we found that the proportions of co-expressed intrachromosomal colocalized gene pairs that were not linear neighbors, always-neighboring gene pairs without intrachromosomal colocalization, evolutionary neighboring gene pairs without intrachromosomal colocalization and no colocalization gene pairs were 7.11, 7.41, 6.64 and 4.5%, respectively (P < 10^−5^, Fig. 1b). Random experiments produced frequencies of co-expressed gene pairs significantly lower than those of the actual colocalized gene pairs. These results indicate that the three kinds of colocalized gene pairs, including spatial colocalization and linear neighbors, all tend to show co-expression. In addition, the always-neighboring gene pairs had the strongest co-expression, intrachromosomal colocalized gene pairs had weakest co-expression among three kinds of colocalized gene pairs. . In terms of threshold of 0.1, we found that the proportions of co-expressed of three types colocalized genes and no colocalization genes were 40.52, 43.64, 42.73 and 38.2%, respectively (P < 10^−5^, Additional file 4: Figure S1A). Random experiments indicate the statistical significance. These results further indicate that in the condition of high correlation or low correlation, always neighboring genes had the strongest co-expression, while intrachromosomal colocalized gene pairs had the weakest co-expression, among three types of colocalized gene pairs, which probably indicate that co-expression is more driven by linear colocalization rather than physical proximity.

Next, we used genome-wide histone modification data from *Arabidopsis thaliana* to compute the absolute difference of the modification level of 16 histones in the three kinds of colocalized gene pairs and no colocalization gene pairs. We found that all three kinds of colocalized gene pairs tended to show similar modification levels of two histones, H3K27me3 and X5mC, the corresponding absolute differences of modification level in intrachromosomal colocalized gene pairs, always-neighboring gene pairs, evolutionary neighboring gene pairs and no colocalization gene pairs were 0.655, 0.646, 0.831, 0.68 (P < 10^−5^, Fig. 1c2) and 0.06, 0.054, 0.073 0.107 (P < 10^−5^, Fig. 1c1), respectively., statistical significance is confirmed by random experiments. In particular, the absolute difference of the X5mC modification level in the three colocalized gene pairs was less than 0.1, which indicates that colocalized gene pairs have extremely similar levels of X5mC modification. What’s more, the absolute differences of H3K27me3 and X5mC modification level in always neighboring and intrachromosomal colocalized genes were consistently smaller than no colocalization genes. These results indicate that: (1) having similar histone modifications is an intrinsic feature of colocalized gene pairs; (2) always-neighboring gene pairs have the strongest similarity, while evolutionary neighboring gene pairs have the weakest similarity, in histone modification level among three kinds of colocalized gene pairs.

Finally, we investigated whether colocalized gene pairs tend to show conservation across species. Hihger conservation of gene pairs across species indicate that they have experienced more similar selection pressures. We computed the frequency of the three kinds of colocalized gene pairs and no colocalized genes in other 20 species, and found that the average frequency of intrachromosomal colocalized gene pairs, always neighboring gene pairs, evolutionary neighboring gene pairs and no colocalized gene pairs is 1.87, 3.02, 4.16 and 1.53 respectively, which are more common than would be expected by chance and no colocalization genes; this was especially true of evolutionary neighboring gene pairs (*P* < 10^−5^, Fig. 1d). These results indicate that colocalized gene pairs, whether linearly or spatially colocalized, have probably experienced more of the same selection pressure in evolution process than no colocalized gene pairs. Moreover, evolutionary neighboring gene pairs showed the greatest conservation, while intrachromosomal colocalized gene pairs showed the weakest conservation, in contrast to the co-expression and co-modification results, which further suggests that some pressures caused by natural selection in *Arabidopsis thaliana* favor chromosomal rearrangements in which separated genes become neighbors in order to better adapt to the environment.

In one word, we found that different types of colocalized genes differentially contribute to their co-expression, co-modification and conservation across species. In terms of co-expression, always-neighboring factor shows the strongest effect, while intrachromosomal colocalization factor shows the weakest effect. In terms of co-modification levels of H3K27me3 and X5mC, always-neighboring factor has the strongest effect and evolutionary neighboring factor has the weakest effect. In terms of conservation across species, evolutionary neighboring factor has the strongest effect, while intrachromosomal colocalization factor shows the weakest effect.

### Intrachromosomal colocalization strengthens the co-expression of always-neighboring and evolutionary neighboring genes

We also investigated whether the superposition of multiple colocalizations can strengthen the co-expression of gene pairs and how strong this effect might be.

**Fig. 2:**
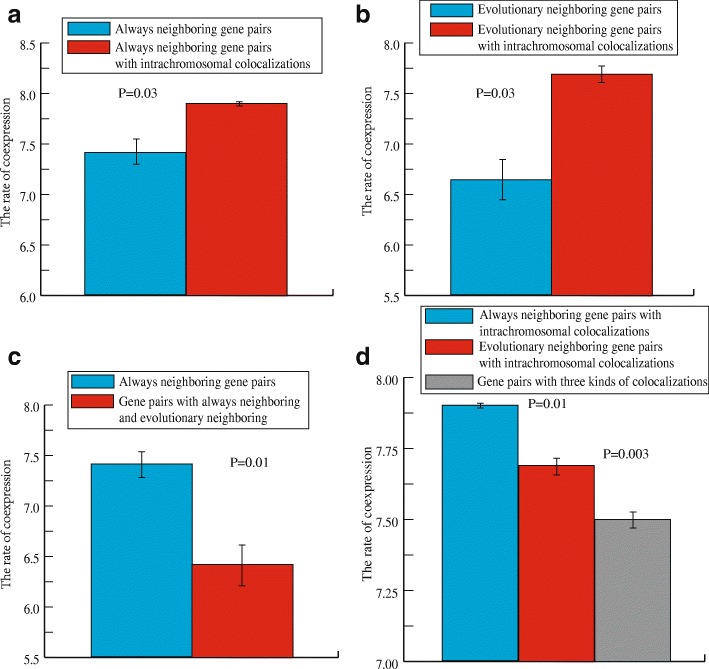
Intrachromosomal colocalization strengthens the co-expression of always-neighboring and evolutionary neighboring genes in threshold 0.5. **a** The rate of co-expression of always-neighboring gene pairs and always-neighboring gene pairs with intrachromosomal colocalization. **b** The rate of co-expression of evolutionary neighboring gene pairs and evolutionary neighboring gene pairs with intrachromosomal colocalization. **c** The rate of co-expression of always-neighboring gene pairs and gene pairs with both always-neighboring and evolutionary neighboring relationships. **d** The rate of co-expression gene pairs with different combinations of colocalizations. Error bars were calculated by bootstrapping. Significance values calculated from the Mann–Whitney U test are shown

First, we computed the Pearson correlation coefficient of always-neighboring gene pairs with intrachromosomal colocalization (1252 pairs, Additional file 5: Table S4) using threshold 0.5 and 0.1 respectively. For 0.5, we found that the percentage of co-expression was 7.9%, which is larger than the co-expression level in always-neighboring gene pairs without intrachromosomal colocalization 7.41%, with a relative increment 6.2% (*P* < 0.05, Mann–Whitney *U* test, Fig. 2a). For 0.1, we found that the percentage of co-expression was 45.76%, larger than the co-expression level in always-neighboring gen pairs without intrachromosomal colocalization 43.64% (P < 0.05, Mann–Whitney *U* test, Additional file 4: Figure S1B), with a relative increment 4.6%. These results indicate that intrachromosomal colocalization can strengthen the co-expression of always-neighboring gene pairs.

Second, we investigated whether intrachromosomal colocalization can strengthen the co-expression of evolutionary neighboring gene pairs. Similar, for threshold 0.5, the frequency of co-expression of evolutionary neighboring gene pairs with intrachromosomal colocalization (389 pairs, Additional file 6: Table S5) was 7.69%, larger than that of evolutionary neighboring without intrachromosomal colocalization 6.64% with a relative increment 13.65% **(***P* < 0.05, Mann–Whitney *U* test, Fig. 2b**)**. For threshold 0.1, the frequency of co-expression of corresponding item was 44.55%, larger than that of single evolutionary neighboring 42.73% with a relative increment 4.1% (*P* < 0.05, Mann–Whitney *U* test, Additional file 4: Figure S1C). These results indicate that intrachromosomal colocalization also strengthens the co-expression of evolutionary neighboring gene pairs.

Third, we investigated whether evolutionary neighboring colocalization further strengthens the co-expression of always-neighboring gene pairs. For threshold 0.5, we found that the percentage of co-expressed gene pairs with both co-localizations (5489 pairs, Additional file 7: Table S6) was 6.42%, smaller than that of gene pairs that were solely always-neighboring 7.41% with a relative decrement − 13.63% (*P* < 0.05, Mann–Whitney *U* test, Fig. 2c). For threshold 0.1, we found that the percentage of co-expressed gene pairs with both co-localizations was 43.18%, smaller than that of genes that were solely aways-neighboring 43.64% with a relative decrement − 1.1% (*P* < 0.05, Mann–Whitney *U* test, Additional file 4: Figure S1D). These results indicate that evolutionary colocalization weakens the co-expression of always-neighboring gene pairs, which suggests that evolutionary colocalization caused by natural selection cannot improve the co-expression of always-linear neighboring gene pairs.

Finally, we investigated whether the combination of the three kinds of colocalization leads to the greatest co-expression. We computed the Pearson correlation coefficient of gene pairs with all three kinds of colocalization (339 pairs, Additional file 8: Table S7). Using threshold 0.5, we found 7.5% co-expression, which is smaller than the co-expression of always-neighboring genes with intrachromosomal colocalization 7.9% and of evolutionary neighboring genes with intrachromosomal colocalization 7.69% (*P* < 0.05, Mann–Whitney *U* test, Fig. 2d). Using threshold 0.1, we also found 44.64% co-expression, which is also consitantly smaller than the co-expression of always-neighboring genes with intrachromosomal colocalization 45.76% and of evolutionary neighboring genes with intrachromosomal colocalization 44.65% (P < 0.05, Mann–Whitney *U* test, Additional file 4: Figure S1E). These findings indicate that the three kinds of colocalization together do not generate the greatest co-expression, further confirming that evolutionary neighboring colocalization and always-neighboring colocalization probably act to co-express neighboring genes through mutually inhibitory mechanisms.

Overall, we found that intrachromosomal colocalization can consistently strengthen the co-expression of neighboring genes, including always-linear neighboring and evolutionary neighboring relationships. This is in contrast to previous suggestions that selectively favorable colocalization in evolution cannot favor the co-expression of neighboring genes and instead acts to constrain the co-expression of always linear colocalization genes. In fact, evolutionary colocalization and always-neighboring colocalization probably act through mutually inhibitory mechanisms to co-express neighboring genes.

### Intrachromosomal colocalization strengthens the co-modification of always-neighboring with linear colocalization and of evolutionary neighboring genes

We also investigated whether the combination of multiple colocalizations strengthens the similarity in the histone modification levels of gene pairs. We computed the absolute difference of the modification level of 16 histones between the gene pairs. Our results indicate that intrachromosomal colocalizationgreatly strengthens the similarity of neighboring genes, including always-neighboring and evolutionary neighboring relationships, in modification level of two histones, H3K27me3 and X5mC.

**Fig. 3:**
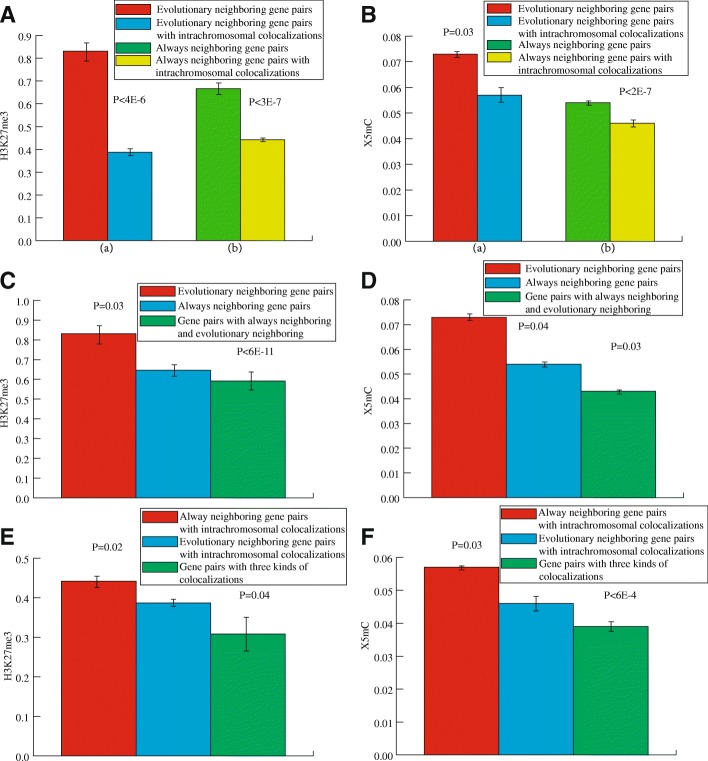
The absolute difference in the modification levels of histones H3K27me3 and X5mC. **a**, **b** Intrachromosomal colocalization strengthens the co-modification of H3K27me3 and X5mC in evolutionary neighboring gene pairs and always-neighboring gene pairs. **c**, **d** Evolutionary neighboring and always-neighboring relationships cause mutual enhancement in the modification levels of histones H3K27me3 and X5mC. **e**, **f** The similarities in the histone modification levels of different combinations of colocalizations. Error bars were calculated by bootstrapping. Significance values calculated from the Mann–Whitney U test are shown

First, we computed the absolute difference in histone modification levels of two histones, H3K27me3 and X5mC, in always-neighboring genes with intrachromosomal colocalization (0.442 and 0.046) and in evolutionary neighboring genes with intrachromosomal colocalization (0.378 and 0.057). We found that intrachromosomal colocalization can greatly strengthen the similarity in the histone modification level of two histones, H3K27me3 and X5mC, in both always-neighboring genes (0.646 and 0.054) and evolutionary neighboring genes (0.831 and 0.073) (*P* < 0.05, Mann–Whitney *U* test, Fig. 3A, B). Specifically, the increase in similarity in the modification level of histone H3K27me3 in always-neighboring genes and evolutionary neighboring genes by intrachromosomal colocalization was 32 and 53.5%, respectively (*P* < 0.05, Mann–Whitney *U* test, Fig. 3A(a, b)), while the corresponding increase for histone X5mCwas 15 and 22% (P < 0.05, Mann–Whitney *U* test, Fig. 3B(a, b)), respectively.

Second, we computed the modification level of H3K27me3 and X5mC in gene pairs with both always-neighboring and evolutionary neighboring colocalizations (0.592 and 0.043). We found that the similarity of histone modifications in these gene pairs was higher than always neighboring gene pairs (0.646 and 0.054) and evolutionary neighboring gene pairs (0.831 and 0.073). The corresponding increasing ratio are 8.4 and 28.8% (P < 0.05, Mann–Whitney *U* test, Fig. 3C) in histone H3K27me3, 20.1 and 41.1% (P < 0.05, Mann–Whitney *U* test, Fig. 3D) in histone X5mC, respectively. These results suggest that neighboring relationship caused by natural selection probably facilitates the modification of histones H3K27me3 and X5mC in gene pairs.

Third, we computed the modification levels of histones H3K27me3 and X5mC in gene pairs with all three kinds of colocalization (0.308 and 0.039) and found that the absolute difference in histone modification levels was at a minimum for both histones. The absolute difference in histone modification levels in gene pairs with all three colocalizations was consistently lower than that of any combination of two colocalizations, such as always-neighboring genes with intrachromosomal colocalization (0.442 and 0.046), evolutionary neighboring with intrachromosomal colocalization (0.378 and 0.057), and the combination of always-neighboring and evolutionary neighboring (0.592 and 0.043). The corresponding increasing ratios of similarities in histone modification levels are 30.3, 20.4, 48% (*P* < 0.05, Mann–Whitney *U* test, Fig. 3E) in the histone H3K27me3 and 18, 31.6, 9.3% (P < 0.05, Mann–Whitney *U* test, Fig. 3F) in the histone X5mC, respectively. These results suggest that gene pairs with all three colocalizations have the greatest similarity in histone co-modification levels, indicating that it is possible to increase the similarity in histone co-modification levels by maximizing gene colocalization both spatially and linearly.

### Intrachromosomal colocalization strengthens the conservation of always-neighboring gene pairs and evolutionary neighboring gene pairs across species

We investigated whether the superposition of multiple colocalizations strengthens the conservation of gene pairs across species. Our findings indicate that intrachromosomal colocalization strengthens the conservation of always-neighboring and evolutionary neighboring gene pairs.

**Fig. 4:**
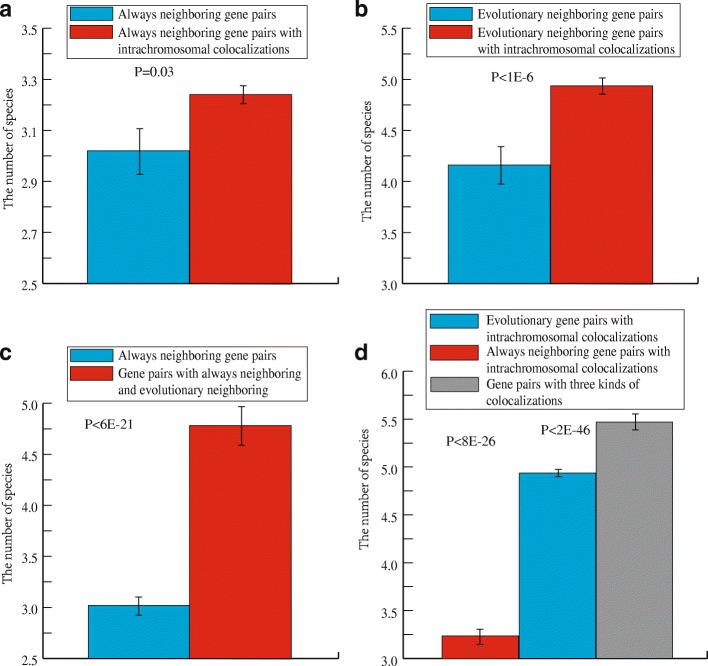
Intrachromosomal colocalization strengthens the conservation of neighboring gene pairs. **a** The number of species in which always-neighboring gene pairs and always-neighboring gene pairs with intrachromosomal colocalization were found. **b** The number of species in which evolutionary neighboring gene pairs and evolutionary neighboring gene pairs with intrachromosomal colocalization were found. **c** The number of species in which always-neighboring gene pairs and always-neighboring and evolutionary neighboring gene pairs were found. **d** The number of species with different combinations of colocalizations. Error bars were calculated by bootstrapping. Significance values calculated from the Mann–Whitney U test are shown

We first computed the average frequency of always-neighboring gene pairs with intrachromosomal colocalization and evolutionary neighboring gene pairs with intrachromosomal colocalization in other 20 species. We found frequencies were 3.24 and 4.94, which is larger than observed for always-neighboring (3.02) and evolutionary neighboring gene pairs (4.16) alone, with an increase of 6.8 and 15.6%, respectively (*P* < 0.05, Mann–Whitney *U* test, Fig. 4a, b). These results indicate that intrachromosomal colocalization strengthens the conservation of neighboring gene pairs across species, especially in the case of evolutionary neighboring gene pairs.

We next investigated whether evolutionary colocalization strengthens the conservation of neighboring gene pairs across species. We found that the average frequency of gene pairs in the other 20 species was 4.78, which is 58.3% higher than that of always-neighboring gene pairs (3.02) (*P* < 0.05, Mann–Whitney *U* test, Fig. 4c). This indicates that (1) evolutionary colocalization caused by natural selection has a greater strengthening effect on the conservation of always-neighboring gene pairs than intrachromosomal colocalization; (2) more species are likely to experience similar environmental pressure which drive gene pairs to be neighbors in evolution.

Finally, we investigated whether the combination of the three kinds of colocalization leads to the greatest conservation of gene pairs. We computed the frequency of gene pairs with the three colocalizations in the other 20 species and found an average frequency of 5.47 (*P* < 0.05, Mann–Whitney *U* test, Fig. 4d), which is 81.1 and 31.5% higher than that of always-neighboring and evolutionary neighboring relationships alone, respectively, and 68.8 and 11% higher than the combination of intrachromosomal colocalization with always-neighboring and evolutionary neighboring relationships, respectively. These findings indicate that gene pairs with all three colocalizations are the most strongly conserved across species, which probably suggest that gene pairs with all three colocalizations have experienced the most similar selection pressure in evolution.

Overall, we found that intrachromosomal colocalization can greatly increase the conservation of neighboring gene pairs across species, including always-neighboring genes and evolutionary neighboring genes, which indicate that neighboring gene pairs with intrachromosomal colocalization have experienced more similar seletion pressure than always-neighboring and evolutionary neighboring gene pairs alone. Furthermore, evolutionary colocalization strengthens the conservation of neighboring gene pairs across species, which further indicates that natural selection favors separated genes becoming neighbors.

## Discussion

### Colocalized gene pairs are indicative of phylogenetic relationships and WGD events

**Fig. 5:**
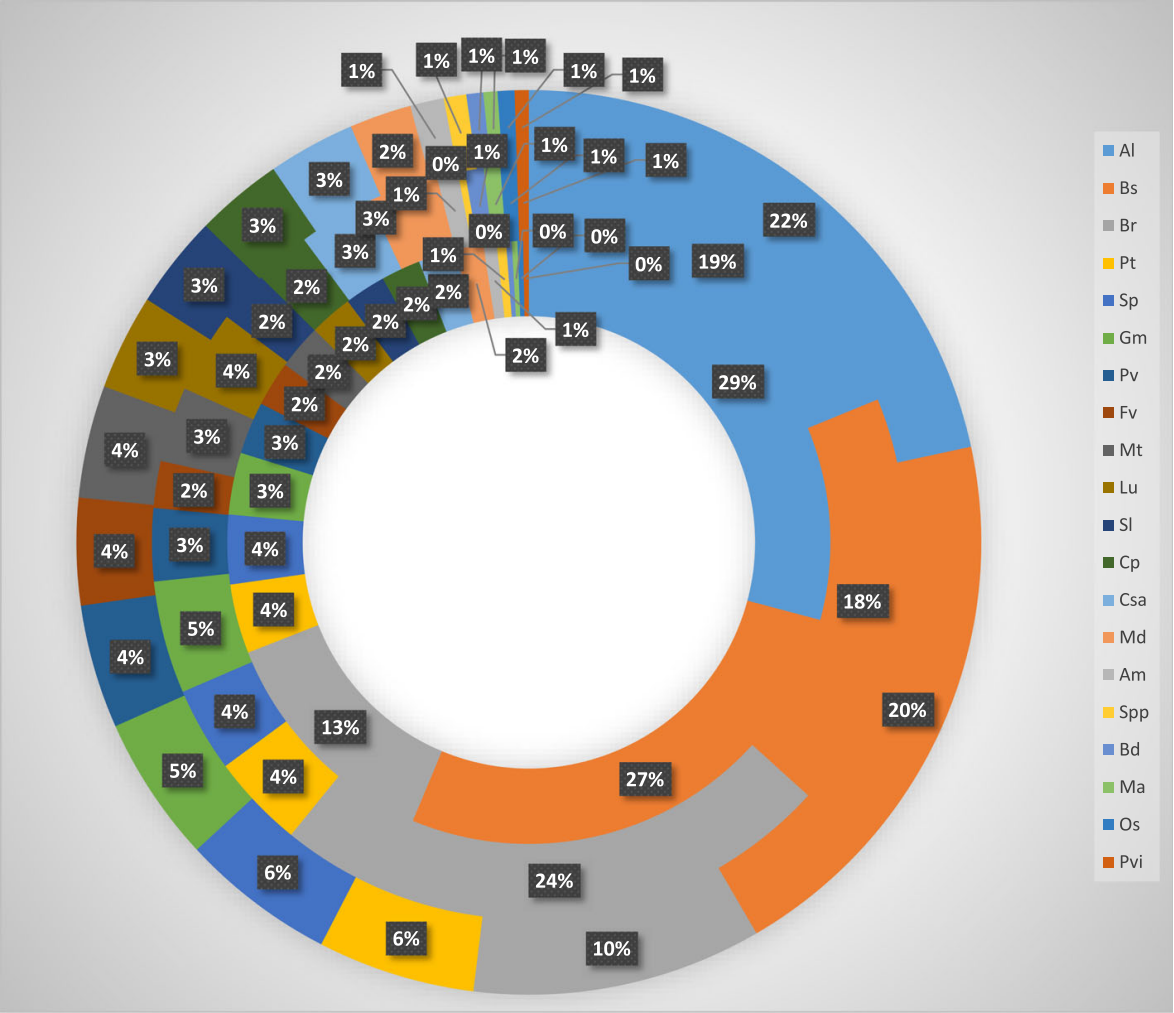
The proportion of the three kinds of colocalized gene pairs found in the other 20 species. From the inner circle to the outer circle are the results of always-neighboring gene pairs, intrachromosomal colocalized gene pairs, and evolutionary neighboring gene pairs

We investigated the phylogenetic relationship of three kinds of colocalized gene pairs in 20 species and found the following: (1) colocalized gene pairs tend to occur in species which have recently experienced whole genome duplications (WGD) events, such as *Populus trichocarpa, Salix purpurea, Glycine max, Phaseolus vulgaris, Fragaria vesca* and *Malus domestica*; (2) the three kinds of colocalized gene pairs are all more common in species with closer phylogenetic relationships, such as *Arabidopsis lyrata*, *Boechera stricta*, and *Brassica rapa* (Fig. 5), which indicates that colocalized gene pairs can reveal phylogenetic relationships. Furthermore, the proportion of colocalized gene pairs in three closely phylogenetic species, *Arabidopsis lyrata*, *Boechera stricta*, and *Brassica rapa*, is consistently larger than the sum in the other 17 species (Fig. 5), which indicates that phylogenetic relationship has a major effect on which genes are colocalized in different species. We fitted a mathematical model of phylogenetic relationships using the always-neighboring gene pairs among the 20 species as follows:

$$ r={r}_1\times {r}_2 $$

where *r*is a fitted phylogenetic coefficient, *r*_1_ = 2*N*_*n*_/*N*_*A*_which is a direct phylogenetic factor and *r*_2_ = *T*_*c*_/*T*_*A*_ is a genome amplification factor, with *N*_*n*_being the number of always neighboring gene pairs, *N*_*A*_ the total gene number of *Arabidopsis thaliana*, *T*_*c*_ the total genome size of the compared species, and *T*_*A*_the total genome size *Arabidopsis thaliana*. Using this mathematical model, we fitted the phylogenetic coefficient of *Arabidopsis thaliana* with the other 20 species (Additional file 9: Table S8). The inferred phylogenetic relationship is consistent with previously published results [27], further confirming that colocalized gene pairs are indicative of phylogenetic relationships and WGD events.

### The co-expressed gene pairs tend to show co-modifications and phylogenetic preference

We tested whether co-expressed colocalized gene pairs tend to have similar histone modification levels and found that this was true of two histones, H3K27me3 and X5mC (Additional file 10: Figure S2). The average modification level of co-expressed gene pairs is 0.629 in H3K27me3 and 0.054 in X5mC, which is much smaller than in randomized experiments (Fig. 1c1, c2). These findings suggest that a significant proportion of the co-expression of colocalized genes might be driven by the distribution of histone modifications. Next, we investigated whether co-expressed gene pairs tend to be phylogenetically close by computing how well they were conserved across species. We found that all three kinds of colocalized gene pairs tended to show strong phylogenetic conservation (Additional file 11: Figure S3), and co-expressed gene pairs were mainly found on phylogenetically close species, such as *Arabidopsis lyrata*, *Boechera stricta*, and *Brassica rapa*, which is consistent with the phenomenon of colocalized gene pairs. These results may indicate that the phylogenetic links between co-expressed gene pairs was caused by their colocalization.

### Conservation is negatively correlated with phylogenetic relationship

We investigated whether there is a relationship between phylogenetic relationships and the conservation of colocalization of gene pairs between species. To do this, we computed the frequency of seven kinds of colocalized gene pairs (Table 1) with different conservation in the 20 species and analyzed the proportions in three phylogenetically close species, *Arabidopsis lyrata*, *Boechera stricta*, and *Brassica rapa*, and other 17 speices. Our results show that the proportion of colocalized gene pairs in three phylogenetically close species decreases with an increase in conservation across species (Additional file 12: Figure S4), which indicates that species conservation of colocalized gene pairs is inversely related to the phylogenetic relationship. This phenomenon probably indicates that gene pairs with high levels of conservation are involved in fundamental biological processes or have primary molecular functions. To address this question, we performed functional enrichment analyses of gene pairs with the highest conservation levels using the web-based tool agriGO [30]. The results demonstrated that gene pairs with the three kinds of colocalization were mainly enriched in functional categories involved in developmental processes such as embryonic and post-embryonic development (GO:0009790, *P* < 8.00E-05), seed development (GO:0048316, *P* < 0.0001), reproductive structure and system development (GO:0022414, *P* < 3.50E-06), cellular binding functions, such as nucleoside phosphate binding (GO:0000166, *P* < 1.80E-08), organic cyclic compound binding (GO:0097159, *P* < 0.0006), and protein binding (GO:0005515, P < 1.80E-05), all of which are primary biological processes and functions.

**Table 1:** The gene pairs identified in this research

Gene pairs	Numbers
Intrachromosomal colocalization without being linear neighbors	490
Always-neighboring without intrachromosomal colocalization	16,456
Evolutionary neighboring without intrachromosomal colocalization	6205
Always-neighboring with intrachromosomal colocalization	1252
Evolutionary neighboring with intrachromosomal colocalization	389
Both always-neighboring and evolutionary neighboring	5489
All three kinds of colocalization	339

### Biological and molecular functions analysis of colocalized gene pairs

To understand the biological significance of colocalized gene pairs, we used GO categorization analysis to determine their biological and molecular function enrichment. The categories regulation of transcription, response to stimulus, and transport were highly enriched in always-neighboring and evolutionary neighboring gene pairs, while post-embryonic development and peptide transport proteins were enriched in intrachromosomal colocalization gene pairs (Additional file 13: Figure S5). In terms of molecular function, over-represented GO categories including DNA binding and hydrolase activity were found in all three kinds of colocalized gene pairs (Additional file 14: Figure S6). However, the always-neighboring and evolutionary neighboring gene pairs were also enriched in genes related to transcription factor activity and protein serine/threonine kinase activity. These results suggest that these genes may tend to coevolve and display similar functions. In addition, 268 common genes were identified in the three kinds of colocalized gene pairs, and many protein serine/threonine phosphatases were enriched (Additional file 15: Table S9). This suggests that the neighboring pattern of these phosphatases is important for their role in growth and development. Altogether, these data suggest that different kinds of proteins tend to display different colocalization patterns which may be related to their different functions in plant development.

## Conclusions

In this study, we performed a comprehensive analysis of the characteristics of three kinds of colocalized gene pairs, intrachromosomal colocalization, always-neighboring, and evolutionary neighboring, including their expression profiles, histone modification levels, conservation across species, and the relationships between each of these traits. First, we investigated the intensity relations in expression profiles, histone modification levels, and conservation of the three kinds of colocalized gene pairs. Our results indicate that although neighboring gene pairs, including linear neighbors and physical neighbors, tend to show co-expression, similar histone modifications, and conservation across species, their intensities are significantly different. Specifically, in terms of experssion profiles, always-neighboring genes and intrachromosomal colocalized genes have strongest and weakest co-expression respectively; in terms of histone modifications, always neighboring genes and evolutionary neighboring genes have strongest and weakest co-modifications respectively; in terms of conservation across species, evolutionary neighboring genes and intrachromosomal colocalized genes have strongest and weakest conservation across species respectively.

Next, we investigated whether the combination of several colocalizations can strengthen or weaken the similarities in gene pairs. Our findings indicate that (1) for co-expression, intrachromosomal colocalization can consistentaly strengthen always-neighboring genes and evolutionary neighboring genes, but evolutionary neighboring weakens the co-expression of always-neighboring genes rather than strengthening it. In this situation, gene pairs with all three kinds of colocalization have not show the strongest co-expression; (2) for co-modification and conservation across species, intrachromosomal colocalization can consistently strengthen always neighboring genes and evolutionary neighboring genes. What’s more, evolutionary neighboring factor also can greatly strengthen the similarities of always-neighboring genes. In this situation, gene pairs with all three kinds of colocalization tend to show the strongest similarity in histone modification levels and conservation. These results probabley suggest that (1) evolutionary neighboring and always-neighboring present a mutually restrictive mechanism controlling gene expression; (2) histone modification and natural selection favor chromosomal rearrangement in which separated genes become neighbors to better adapt to external environments.

Finally, we investigated the relationship between co-expression, co-modification, conservation, and the phylogenetic relationship of colocalized gene pairs and found that: (1) Co-expressed gene pairs with colocalization tend to have similar histone modification levels; (2) Colocalized gene pairs are indicative of phylogenetic relationships and WGD events; (3) Conservation across species is negatively correlated with phylogenetic relationships. These results provide new insights into the co-expression of colocalized genes in evolution.

Taken together, our results show that colocalized gene pairs, including linearly colocalized and spatially colocalized genes, tend to have different intensities of similarities in terms of co-expression, co-modification, and conservation across species. Furthermore, appropriate combinations of colocalization can strengthen these similarities, but inppropriate combinations of them can weaken their similarities. These results provide valuable clues for future efforts to understand the characteristics of how the neighborhood of genes affects their interactions and functions.

## Methods

### Identification of phylogenetic species and Ortholog analysis

We used the model species *Arabidopsis thaliana* because of the tremendous amount of molecular data available, especially intrachromosomal 3D colocalization data. We also used 20 other species, including 14 eudicots, 5 monocots, and the basal angiosperm *Amborella trichopoda*, to search for orthologous gene pairs based on phylogenetic relationships and WGD events. According to the phylogenetic tree of 21 speices, other 20 species were considered to be the ancestral state that is the socalled evolutionary past for *Arabidopsis thaliana.* We used the orthology analysis software InParanoid 7 [31] with default parameters to search for orthologous gene pairs between *Arabidopsis thaliana* and the other 20 species, and then determined the order of all orthologous gene pairs including their locations and neighborhood relationships using gene annotation data of 21 species.

### Identification of gene order from orthologous gene pairs

By analzing the locations and neighborhood relationships of orthologous gene pairs, we identified always neighboring gene pairs and evolutionary neighboring gene pairs. Always neighboring gene pairs refer to the gene pairs which are both neighboirng in other 20 species and in *Arabidopsis thaliana.* Evolutionary neighboring gene pairs refer to the gene pairs which are separated in other 20 species but neighboring in *Arabidopsis thaliana.* Next, intrachromosomal colocalized gene pairs were identified by using intrachromosomal interaction data which were taken from [32]. By combining the intrachromosomal interaction data, we identified seven classes of gene pairs with different kinds of colocalization (Table 1 and Additional files 1, 2, 3: Tables S1-S3, Additional files 5, 6, 7, 8, 9: Tables S4-S8, Additional file 15: Table S9). In particular, neighboring gene pairs refer to gene pairs which are linear neighbors in chromosome, separated gene pairs refer to gene pairs are not linear neighbors in chromosome.

### Statistical methods

We used the Mann-Whitney U-test (function ‘ranksum’ in software‘MATLAB’ version R2015b) to examine whether there is statistical significance between given two samples, the default significance level is 0.05. The Mann-Whitney U-test is a nonparametric test for equality of population medians of two independent samples. The main advantage of this test is that it makes no assumption that the samples are from normal distributions. Error bars in figures werecalculated by bootstrapping: Data points in a data set are randomly resampled to create 1000 different data sets (each has the same number of data points as the original data set, function bootstrp’ in software ‘MATLAB’ version R2015b), and the mean value is computed for each data set, and standard deviation is computed for the 1000 mean values.

## Additional files

Additional file 1: **Table S1.** Intrachromosomal colocalized gene pairs which were not linear neighbors. (XLSX 27 kb)

Additional file 2: **Table S2.** Always-neighboring gene pairs without intrachromosomal colocalization. (XLSX 295 kb)

Additional file 3: **Table S3.** Evolutionary neighboring gene pairs without intrachromosomal colocalization. (XLSX 131 kb)

Additional file 4: **Figure S1.** Intrachromosomal colocalization strengthens the co-expression of always-neighboring and evolutionary neighboring genes in threshold 0.1. (A) The red curves show the frequency distributions for 10,000 permuted randomizations of the same number of pairs as in the real data, other four vertical dotted line show the frequency of co-expressed gene pairs of three colocalized gene pairs and no-colocalized genes in threshold 0.1. (B) The rate of co-expression of always-neighboring gene pairs and always-neighboring gene pairs with intrachromosomal colocalization. (C) The rate of co-expression of evolutionary neighboring gene pairs and evolutionary neighboring gene pairs with intrachromosomal colocalization. (D) The rate of co-expression of always-neighboring gene pairs and gene pairs with both always-neighboring and evolutionary neighboring relationships. (E) The rate of co-expression gene pairs with different combinations of colocalizations. Error bars were calculated by bootstrapping. Significance values calculated from the Mann–Whitney U test are shown. (PDF 402 kb)

Additional file 5: **Table.** Always-neighboring gene pairs with intrachromosomal colocalization. (XLSX 34 kb)

Additional file 6: **Table S5.** Evolutionary neighboring gene pairs with intrachromosomal colocalization. (XLSX 15 kb)

Additional file 7: **Table S6.** Gene pairs with both always-neighboring and evolutionary neighboring colocalizations. (XLSX 118 kb)

Additional file 8: **Table S7.** Gene pairs with all three kinds of colocalization. (XLSX 15 kb)

Additional file 9: **Table S8.** The fitted phylogenetic coefficient of *Arabidopsis thaliana* with other 22 species by using always neighboring gene pairs. The last column of Table S8 is the fitted phylogenetic coefficient, the first column is the name of 22 species, the second column is the numuber of always neighboring gene pairs compared with *Arabidopsis thaliana*, the third column is the number of total genes of corresponding species. (DOCX 15 kb)

Additional file 10: **Figure S2.** The histone modification levels of coexpressed gene pairs with three different kinds of colocalizations. (DOCX 73 kb)

Additional file 11: **Figure S3.** The proportion of coexpressed gene pairs occurred in other 22 species. (DOCX 90 kb)

Additional file 12: **Figure S4.** The relationship between number of species in which gene pairs are occurred of colocalized gene pairs and their phylogenetic relationship with others. (DOCX 75 kb)

Additional file 13: **Figure S5.** The biological process enrichment analysis of colocalized gene pairs. (DOCX 18 kb)

Additional file 14: **Figure S6.** The molecular function enrichment analysis of colocalized gene pairs. (DOCX 17 kb)

Additional file 15: **Table S9.** Contians 268 serine and phosphatases enriched common genes. (XLSX 11 kb)
