# Supplementary figures and images for: Intrachromosomal colocalization strengthens co-expression, co-modification and evolutionary conservation of neighboring genes
Source: BMC Genomics. 2018 Jun 13;19:455. doi: 10.1186/s12864-018-4844-1 (PMC6000932; doi:10.1186/s12864-018-4844-1)

A

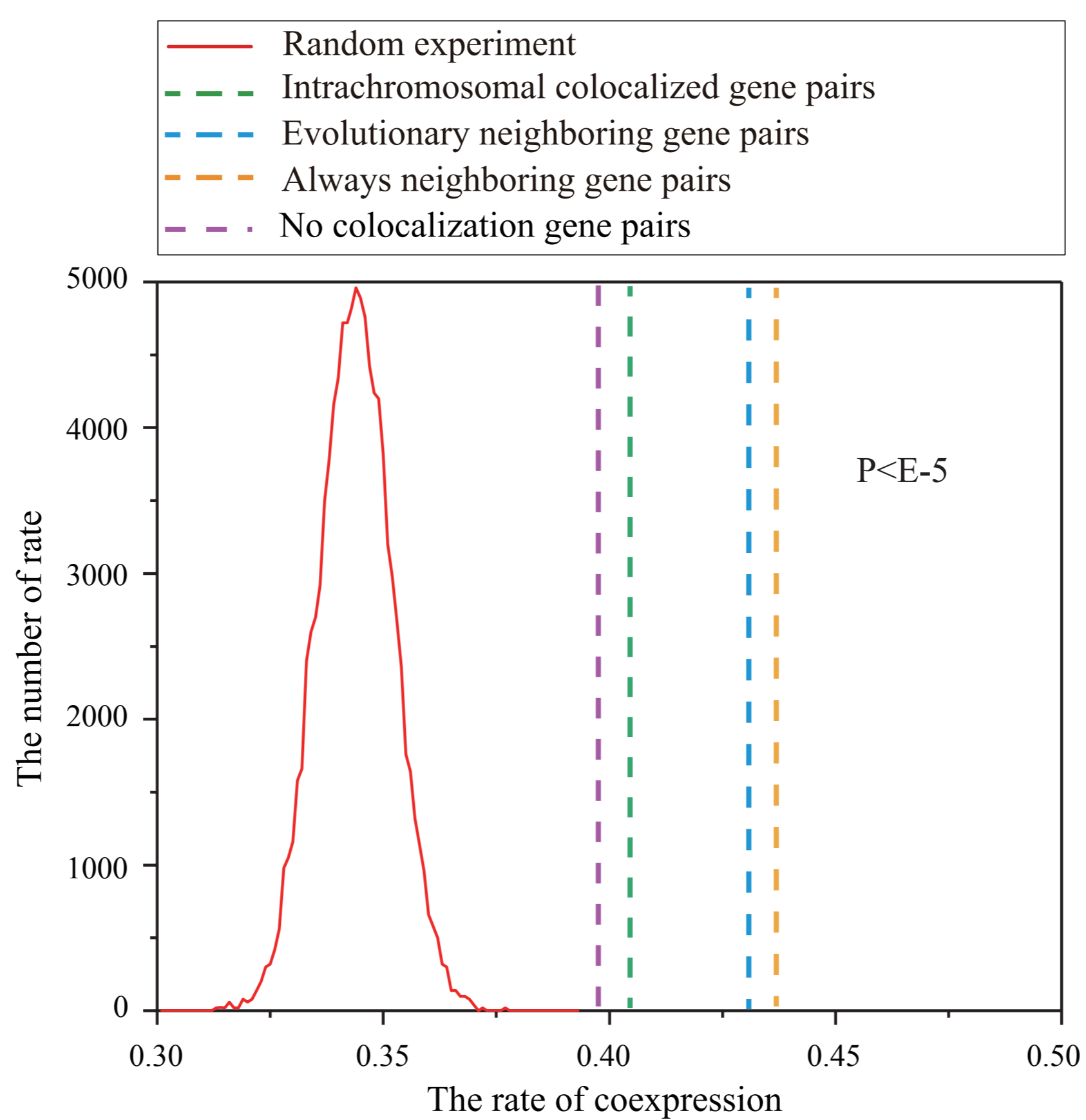

B

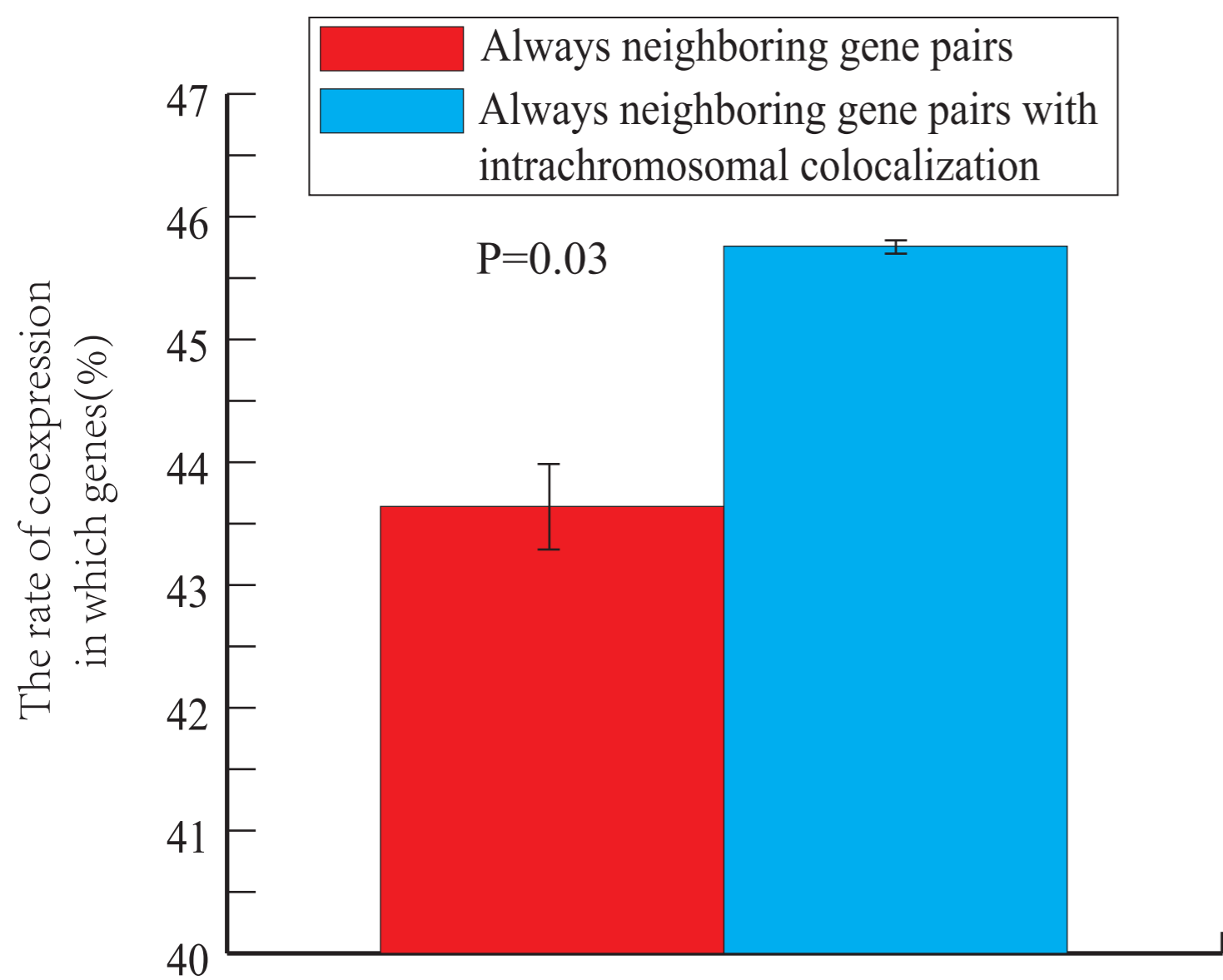

C

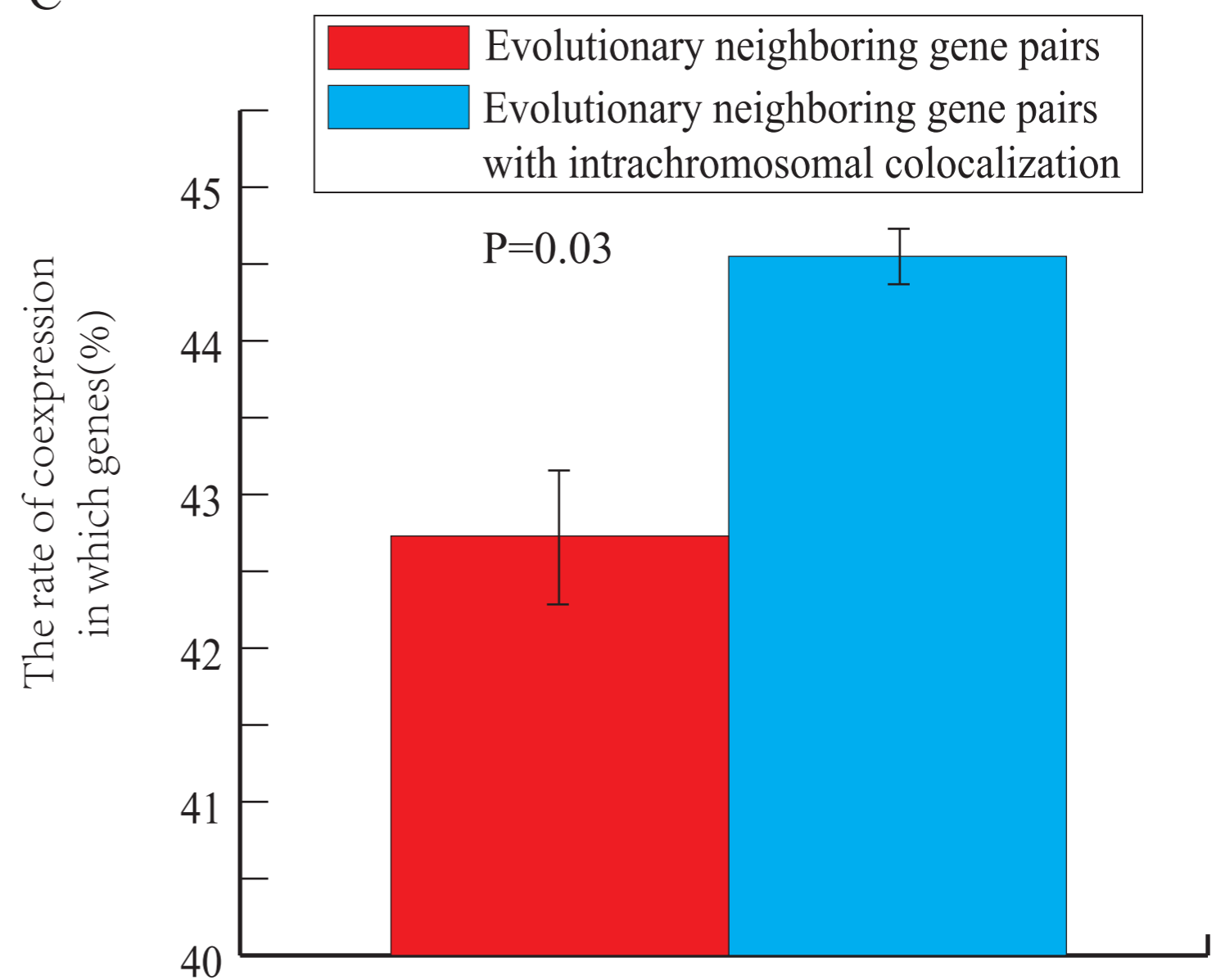

D

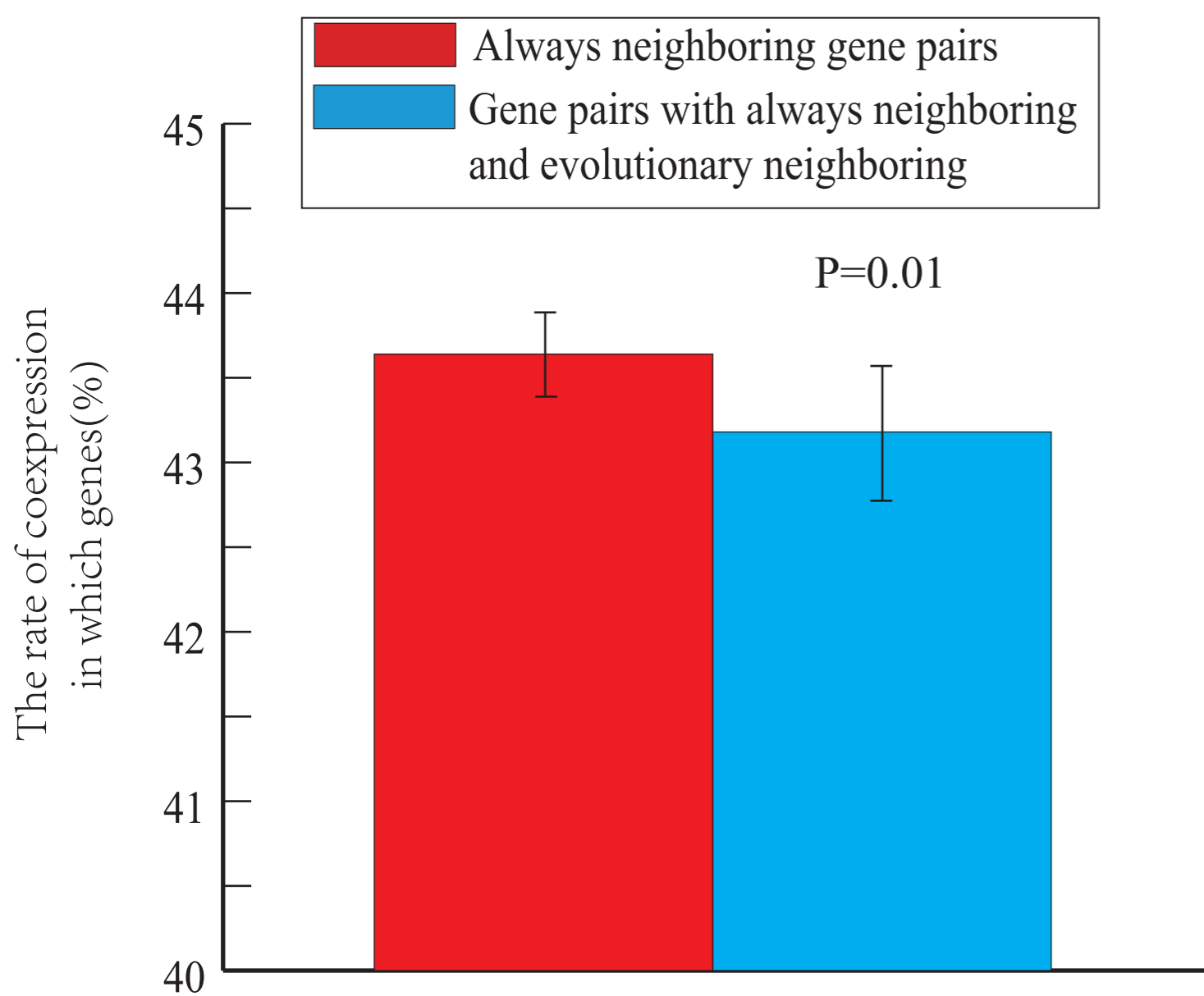

E

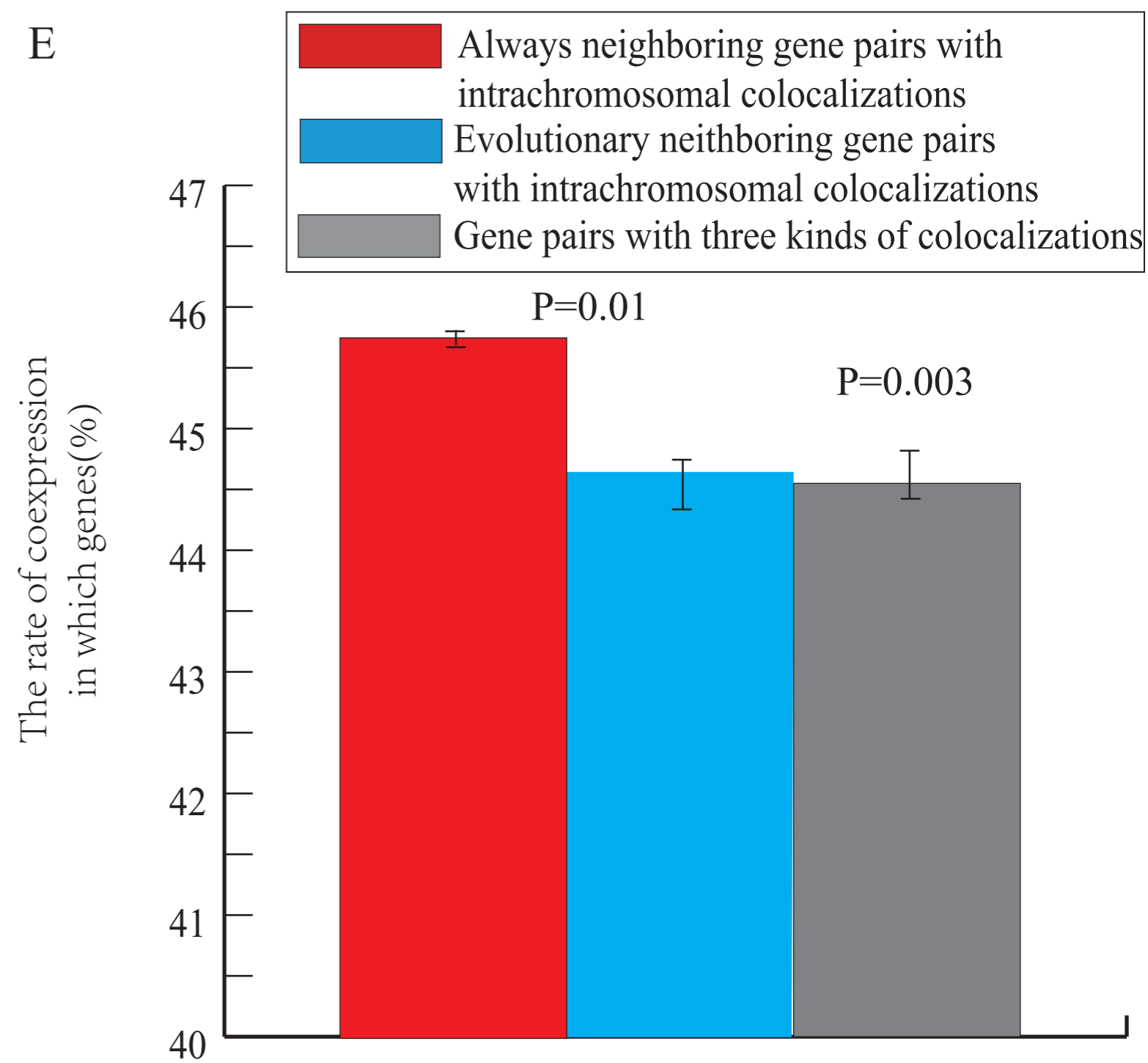

Supplement: Supplementary file 4 — Figure S1. Intrachromosomal colocalization strengthens the co-expression of always-neighboring and evolutionary neighboring genes in threshold 0.1. (A) The red curves show the frequency distributions for 10,000 permuted randomizations of the same number of pairs as in the real data, other four vertical dotted line show the frequency of co-expressed gene pairs of three colocalized gene pairs and no-colocalized genes in threshold 0.1. (B) The rate of co-expression of always-neighboring gene pairs and always-neighboring gene pairs with intrachromosomal colocalization. (C) The rate of co-expression of evolutionary neighboring gene pairs and evolutionary neighboring gene pairs with intrachromosomal colocalization. (D) The rate of co-expression of always-neighboring gene pairs and gene pairs with both always-neighboring and evolutionary neighboring relationships. (E) The rate of co-expression gene pairs with different combinations of colocalizations. Error bars were calculated by bootstrapping. Significance values calculated from the Mann–Whitney U test are shown. (PDF 402 kb) [file 12864_2018_4844_MOESM4_ESM.pdf]
