# Supplementary material for: Intrachromosomal colocalization strengthens co-expression, co-modification and evolutionary conservation of neighboring genes
Source: BMC Genomics. 2018 Jun 13;19:455. doi: 10.1186/s12864-018-4844-1 (PMC6000932; doi:10.1186/s12864-018-4844-1)
Supplement: Supplementary file 9 — Table S8. The fitted phylogenetic coefficient of Arabidopsis thaliana with other 22 species by using always neighboring gene pairs. The last column of Table S8 is the fitted phylogenetic coefficient, the first column is the name of 22 species, the second column is the numuber of always neighboring gene pairs compared with Arabidopsis thaliana, the third column is the number of total genes of corresponding species. (DOCX 15 kb) [file 12864_2018_4844_MOESM9_ESM.docx]

Supplementary table 9. The fitted phylogenetic coefficient of *Arabidopsis thaliana* with other 22 species by using always neighboring gene pairs.

| Species | Always Neighboring | Total Genes | Phylogenetic coefficient |
| --- | --- | --- | --- |
| Arabidopsis lyrata (Al) | 14034 | 31478 | 1.19 |
| Boechera stricta (Bs) | 13642 | 27416 | 1.01 |
| Brassica rapa (Br) | 6449 | 39611 | 0.69 |
| Carica papaya (Cp) | 802 | 27769 | 0.06 |
| Salix purpurea (Sp) | 2004 | 37865 | 0.20 |
| Populus trichocarpa (Pt) | 1701 | 39009 | 0.18 |
| Linum usitatissimum (Lu) | 1486 | 43471 | 0.17 |
| Glycine max (Gm) | 2154 | 55589 | 0.32 |
| Phaseolus vulgaris (Phv) | 1296 | 27179 | 0.09 |
| Medicago truncatula (Mt) | 753 | 54073 | 0.11 |
| Fragaria vesca (Fv) | 836 | 32831 | 0.07 |
| Malus domestica (Md) | 741 | 63514 | 0.13 |
| Cucumis sativus (Cs) | 920 | 21503 | 0.05 |
| Solanum lycopersicum (Sl) | 902 | 33857 | 0.08 |
| Panicum virgatum (Pav) | 145 | 98007 | 0.04 |
| Brachypodium distachyon (Bd) | 169 | 26523 | 0.01 |
| Oryza sativa (Os) | 145 | 38864 | 0.02 |
| Musa acuminata (Ma) | 167 | 33598 | 0.02 |
| Spirodela polyrhiza (Sp) | 231 | 19623 | 0.01 |
| Amborella trichopoda (Am) | 269 | 27329 | 0.02 |
