# Supplementary material for: Intrachromosomal colocalization strengthens co-expression, co-modification and evolutionary conservation of neighboring genes
Source: BMC Genomics. 2018 Jun 13;19:455. doi: 10.1186/s12864-018-4844-1 (PMC6000932; doi:10.1186/s12864-018-4844-1)
Supplement: Supplementary file 11 — Figure S3. The proportion of coexpressed gene pairs occurred in other 22 species. (DOCX 90 kb) [file 12864_2018_4844_MOESM11_ESM.docx]

The proportion of coexpressed gene pairs occurred in other 22 species.
