# Supplementary material for: Intrachromosomal colocalization strengthens co-expression, co-modification and evolutionary conservation of neighboring genes
Source: BMC Genomics. 2018 Jun 13;19:455. doi: 10.1186/s12864-018-4844-1 (PMC6000932; doi:10.1186/s12864-018-4844-1)

The relationship between number of species in which gene pairs are occurred of colocalized gene pairs and their phylogenetic relationship with others.


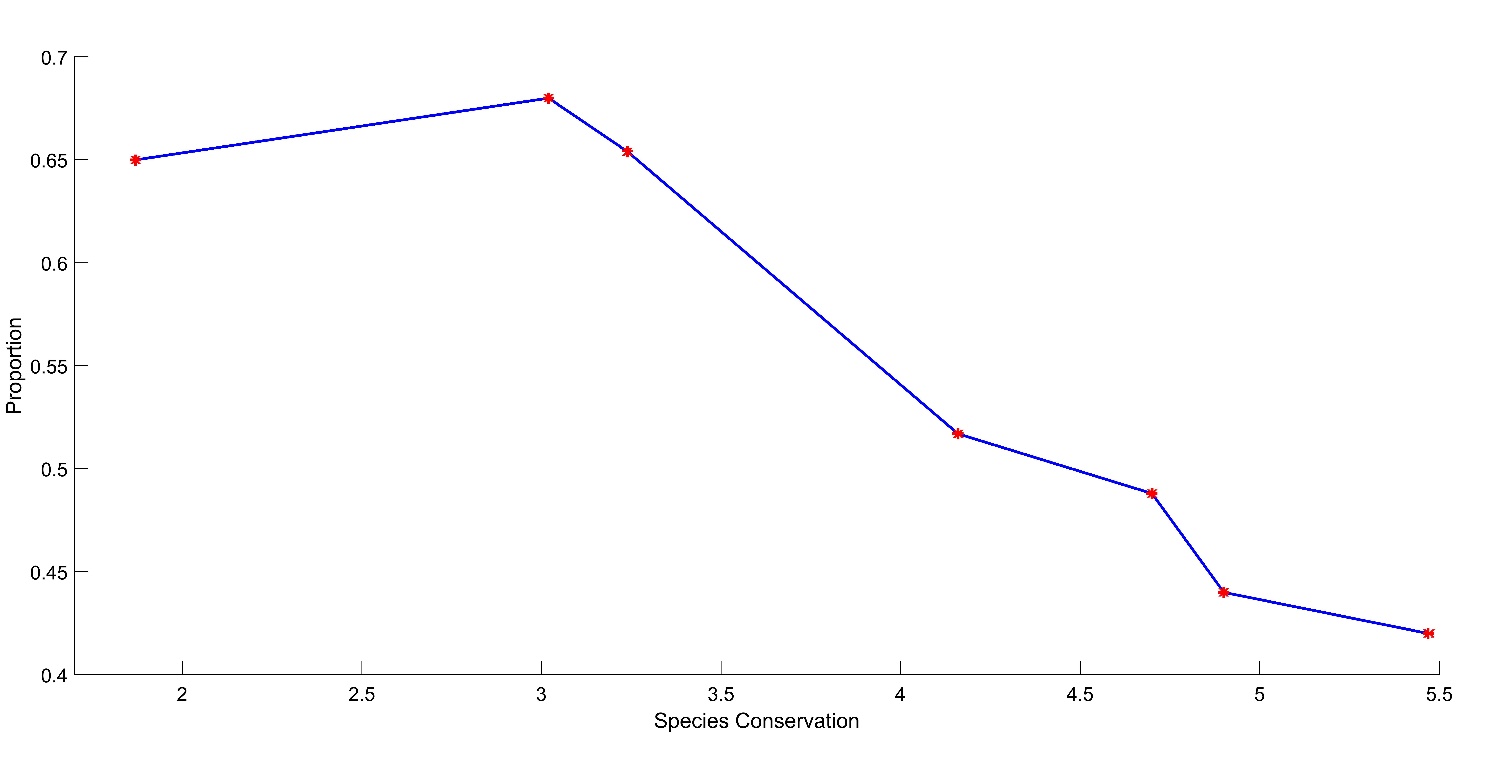

Supplement: Supplementary file 12 — Figure S4. The relationship between number of species in which gene pairs are occurred of colocalized gene pairs and their phylogenetic relationship with others. (DOCX 75 kb) [file 12864_2018_4844_MOESM12_ESM.docx]
