# Supplementary material for: Intrachromosomal colocalization strengthens co-expression, co-modification and evolutionary conservation of neighboring genes
Source: BMC Genomics. 2018 Jun 13;19:455. doi: 10.1186/s12864-018-4844-1 (PMC6000932; doi:10.1186/s12864-018-4844-1)
Supplement: Supplementary file 13 — Figure S5. The biological process enrichment analysis of colocalized gene pairs. (DOCX 18 kb) [file 12864_2018_4844_MOESM13_ESM.docx]

The biological process enrichment analysis of colocalized gene pairs.
